# Proteomics and cytokine analyses distinguish myalgic encephalomyelitis/chronic fatigue syndrome cases from controls

**DOI:** 10.1186/s12967-023-04179-3

**Published:** 2023-05-13

**Authors:** Ludovic Giloteaux, Jiayin Li, Mady Hornig, W. Ian Lipkin, David Ruppert, Maureen R. Hanson

**Affiliations:** 1 Department of Molecular Biology and Genetics, Cornell University, 323 Biotechnology Building, 526 Campus Road, Ithaca, NY 14853 USA; 2 Department of Statistics and Data Science, Cornell University, Ithaca, NY USA; 3 Center for Infection and Immunity, Columbia University Mailman School of Public Health, New York, NY USA; 4 Department of Epidemiology, Columbia University Mailman School of Public Health, New York, NY USA; 5 Departments of Neurology and Pathology, College of Physicians and Surgeons, Columbia University, New York, NY USA; 6 School of Operations Research and Information Engineering, Cornell University, Ithaca, NY USA

**Keywords:** Myalgic encephalomyelitis/chronic fatigue syndrome, Extracellular vesicles, Plasma, Proteomics, Cytokines

## Abstract

**Background:**

Myalgic encephalomyelitis/chronic fatigue syndrome (ME/CFS) is a complex, heterogenous disease characterized by unexplained persistent fatigue and other features including cognitive impairment, myalgias, post-exertional malaise, and immune system dysfunction. Cytokines are present in plasma and encapsulated in extracellular vesicles (EVs), but there have been only a few reports of EV characteristics and cargo in ME/CFS. Several small studies have previously described plasma proteins or protein pathways that are associated with ME/CFS.

**Methods:**

We prepared extracellular vesicles (EVs) from frozen plasma samples from a cohort of Myalgic Encephalomyelitis/Chronic Fatigue Syndrome (ME/CFS) cases and controls with prior published plasma cytokine and plasma proteomics data. The cytokine content of the plasma-derived extracellular vesicles was determined by a multiplex assay and differences between patients and controls were assessed. We then performed multi-omic statistical analyses that considered not only this new data, but extensive clinical data describing the health of the subjects.

**Results:**

ME/CFS cases exhibited greater size and concentration of EVs in plasma. Assays of cytokine content in EVs revealed IL2 was significantly higher in cases. We observed numerous correlations among EV cytokines, among plasma cytokines, and among plasma proteins from mass spectrometry proteomics. Significant correlations between clinical data and protein levels suggest roles of particular proteins and pathways in the disease. For example, higher levels of the pro-inflammatory cytokines Granulocyte-Monocyte Colony-Stimulating Factor (CSF2) and Tumor Necrosis Factor (TNFα) were correlated with greater physical and fatigue symptoms in ME/CFS cases. Higher serine protease SERPINA5, which is involved in hemostasis, was correlated with higher SF-36 general health scores in ME/CFS. Machine learning classifiers were able to identify a list of 20 proteins that could discriminate between cases and controls, with XGBoost providing the best classification with 86.1% accuracy and a cross-validated AUROC value of 0.947. Random Forest distinguished cases from controls with 79.1% accuracy and an AUROC value of 0.891 using only 7 proteins.

**Conclusions:**

These findings add to the substantial number of objective differences in biomolecules that have been identified in individuals with ME/CFS. The observed correlations of proteins important in immune responses and hemostasis with clinical data further implicates a disturbance of these functions in ME/CFS.

**Supplementary Information:**

The online version contains supplementary material available at 10.1186/s12967-023-04179-3.

## Background

Myalgic encephalomyelitis/chronic fatigue syndrome (ME/CFS) is a serious disease that can be diagnosed following 6 months of new debilitating fatigue, post-exertional malaise, unrefreshing sleep, and either or both of two additional symptoms, cognitive difficulty or orthostatic intolerance [1]. Most patients report that their symptoms arose after a viral-like illness, but the identity of the preceding infection is almost always unknown, although the enteroviral family has sometimes been implicated [2, 3]. Before 2020, 65 million individuals world-wide were estimated to experience ME/CFS [4]. Since the SARS-COV2 pandemic, a subset of individuals who had suffered acute COVID-19 have been continuing to experience symptoms [5], and some victims of Long COVID fulfill the ME/CFS diagnostic criteria described above [6]. Likewise, individuals experiencing Gulf War Illness have symptom overlap with both Long COVID and ME/CFS [7]. However, a number of assays, such as neuroimaging [8], distinguish Gulf War Illness and ME/CFS. Whether Long COVID and ME/CFS not associated with SARS-CoV-2 infection will likewise be differentiated through imaging or other measures is not yet known.

Proteins related to the innate immune system and involved in the complement cascade as well as in pathways related to dopamine signaling have been reported to be enriched in ME/CFS patients compared to controls in studies analyzing cerebrospinal fluid [9, 10]. Through plasma mass spectrometry analysis, dysregulations in energy, lipid and amino acid metabolism were also reported in ME/CFS [11–13]. But more recently, a ME/CFS-related plasma proteome analysis using untargeted ultra-performance liquid chromatography-tandem mass spectrometry identified differing profiles between ME/CFS patients, as well as ME/CFS subgroups (with or without IBS), and controls and a set of proteins that may predict ME/CFS status with a reasonably high degree of accuracy (Area Under the Curve (AUC) = 0.774–0.838) [14].

It is known that immune function and inflammatory responses are regulated by cytokines acting as modulators, and that their secretion can occur in classical secretion manner or via encapsulation in extracellular vesicles, protecting them from degrading enzymes [15]. EVs are one of the main participants in cell-to-cell communication and drive inflammatory, autoimmune and infectious disease pathology [16–19] and previous reports have shown increased numbers of circulating EVs, not only in cancers and Alzheimer’s disease [17, 20–22], but also in ME/CFS [23–25]. A recent study on EVs isolated from ME/CFS patients and from subjects with idiopathic chronic fatigue and clinical depression was able to distinguish the two groups with an AUC of 0.802 solely using circulating EV numbers, which allowed a correct diagnosis in 90–94% of ME/CFS cases [24].

Further molecular characterization of ME/CFS is urgently needed to provide insights into the disruptions that occur in the illness. Multi-omic studies performed on the same set of subjects have high potential to provide new hypotheses. Furthermore, being able to distinguish ME/CFS subjects from healthy controls at high sensitivity and specificity would allow monitoring of the effect of experimental therapies. Utilization of blood samples to assess ME/CFS-associated abnormalities would be particularly valuable in comparison to methods that are more invasive or cumbersome.

In this study, we isolated extracellular vesicles (EVs) from blood samples collected prior to 2020 from ME/CFS subjects and heathy controls and measured their cytokine content. This newly generated data along with data already published from a tandem mass-spectrometry plasma proteomic analysis [14] and plasma cytokine levels determination [26] on the same samples were used all together for multiple statistical analysis. We identified a suite of EV cytokines that significantly differ in levels between ME/CFS subjects and controls. We observed correlations between levels of different EV cytokines, between levels of plasma cytokines, between EV cytokines and plasma cytokines, and between cytokines and other plasma proteins. We also detected relationships between plasma cytokines and severity of certain ME/CFS symptoms. In controls, levels of four plasma proteins were related to health measures. A protein involved in hemostasis, SERPINA5, was positively correlated with higher SF-36 function scores. Using machine learning, we identified the 20 proteins with the highest feature importance values. Using these 20 analytes and XGBoost, we could discriminate ME/CFS and controls subjects at an extremely high sensitivity and specificity (AUC = 0.947).

## Methods

### Study population

A sub-population of 49 ME/CFS cases and 49 healthy controls from the Chronic Fatigue Initiative cohort [27] were analyzed in the framework of this current study. All cases met the 1994 CDC Fukuda [28] and/or 2003 Canadian consensus criteria for ME/CFS [29]. On the day of blood collection, clinical symptoms and baseline health status were assessed using the Short Form 36 Health Survey (SF-36) [30] and the Multidimensional Fatigue Inventory (MFI) scale [31]. Peripheral blood was drawn in sodium citrate BD VacutainerTM Cell Preparation Tubes and centrifuged to pellet red blood cells. Resulting plasma samples were received from four locations from supervising physicians as shown: Salt Lake City, Utah (Lucinda Bateman), Incline Village, Nevada (Daniel Peterson), Miami, Florida (Nancy Klimas), and New York City, New York (Susan Levine) and stored at – 80 ℃ and shipped from Columbia University to Cornell University on dry ice and stored at – 80 ℃ prior to processing for isolation of extracellular vesicles. Written consent was obtained from all participants and all protocols were approved by the Institutional Review Board at Columbia University Irving Medical Center.

### Purification of extracellular vesicles

Extracellular vesicles (EVs) were isolated from plasma samples by precipitation using the ExoQuick^™^ reagent (System Biosciences, Palo Alto, CA, USA) as previously described [25]. Briefly, plasma samples from each subject were thawed on ice and centrifuged at 3000 ×*g* for 15 min at room temperature to remove cells and debris. Thrombin (611 U/ml) (System Bioscience, Palo Alto, CA, USA) was added and samples were incubated for 5 min at room temperature to remove fibrinogen, centrifuged at 10,000 ×*g* for 5 min, and the supernatant was collected. The samples were then incubated with ExoQuick^™^ for 60 min at 4 °C, centrifuged at 12,000 ×*g* for 5 min, and the resulting pellet was resuspended in 250 ul of sterile phosphate buffered saline 1X, pH 7.4. Samples were aliquoted for quantification of cytokines/chemokines and growth factors.

### Size and quantification of extracellular vesicles

Concentration and size distribution of isolated EVs were assayed in samples using a NanoSight NS300 instrument (Malvern, Worcestershire, UK) at the Cornell Nanoscale Science and Technology Facility. Samples were thawed and diluted to 1:2000 in PBS 1X and 1 ml was injected through the laser chamber (NanoSight Technology, London, UK). Three recordings of 60-s digital videos of each sample were acquired and analyzed by the NanoSight NTA 2.3 software to determine the size and the concentration of nanoparticles. Results were averaged together.

### Immune profiling of plasma and extracellular vesicles

Immune molecules in plasma were previously measured using a magnetic bead-based 61-plex immunoassay (customized ProcartaTM immunoassay, Affymetrix) [26]. The immune profiling of extracellular vesicles was performed at the Human Nutritional Chemistry Service Laboratory at Cornell University using a human 48-plex magnetic bead kit (Bio-Plex Pro Human Cytokine Screening Panel, 48-plex, Bio-Rad). Prior to analysis, EV samples were treated with Triton 1% to allow the release of encapsulated cytokines [32]. Each sample was measured in duplicate on a MAGPIX^®^ Multiplexing System (Luminex Corp.). For each well, we used the median fluorescence intensity of all beads measured for a given analyte and averaged the two replicates and results were accepted when the coefficient of variation (CV) was below 15%.

### Plasma proteomics

Plasma proteomic profiling was conducted at Columbia University as previously described [14]. Samples from the 49 ME/CFS cases and 49 controls included in this study were run in two batches of 20 samples (11 ME/CFS cases, 9 controls) and 78 samples (38 ME/CFS cases, 40 controls). The 20 samples in the first batch were randomly selected. The cases and controls were frequency-matched on the same matching variables as the total study population. A total of 257 and 279 annotated proteins were measured in the 20 subject sample set and 78 subject sample set, respectively, with an overlap of 207 annotated proteins in both sample sets.

### Statistical analysis

All statistical analyses were performed using R version 4.0.2 (2020-06-22) via RStudio. For each protein analyte, non-detectable values were replaced with half of their minimum value. Protein levels were then log-transformed with base 2 and standardized for further analysis. Z scores and P values were calculated for outlier analysis. The non-parametric Wilcoxon signed-rank tests were performed to test the significance of differences (p < 0.05) between cases and controls for age, BMI, SF-36 survey scores, and EV sizes and concentrations. The robust linear regression was performed using the *rlm* function in the MASS package for determining the significance of differences for each analyte in control and ME/CFS groups with age, BMI, Irritable Bowel Syndrome (IBS), and sex as confounding variables. Robust linear regression was performed to eliminate contamination with outliers or influential observations. Robust linear regression is a form of weighted least squares regression, and we chose M-estimation with Huber weighting [33, 34] for further analysis.

Principal Component Analysis (PCA) was used to simplify the data and increase interpretability by reducing the dimensionality of the protein levels datasets. PCA was performed using the *stats* package in R. Spearman’s rank correlation coefficients were also estimated within protein analytes and between proteins and the metadata (age, BMI, sex, SF-36 scores, IBS). Point-biserial correlations were used when one of the variables was binary (e.g., female vs. male, with vs without IBS). Categorical variables were coded as follows: Cohort: control = 0; ME/CFS = 1; Sex: female = 0; male = 1; IBS: no IBS = 0; with IBS = 1. Throughout, all p values were adjusted for multiple hypotheses using the Benjamini–Hochberg method (FDR) [35, 36].

A machine learning approach was used to identify variables discriminating the two groups of samples (feature selection). Classification of samples as ME/CFS or healthy controls was carried out by using three supervised learning algorithms: random forest [37] implemented using R’s Random Forest function; XGBoost [38] using R’s *xgboost* package and the Least Absolute Shrinkage and Selection Operator (LASSO) penalty [39] applied to logistic regression using the R function *glmnet*. As features, the algorithms used all 353 protein analytes, EV cytokines, plasma cytokines and plasma proteomics. Feature importance for each classifier was calculated. For LASSO, the coefficients of “unimportant” features are shrunk to zero, hence feature importance can be evaluated by “percentage” (out of 250 random resampling cross-validation iterations) in which the predictor’s parameter estimate in the best fitting model is nonzero. For random forest, “Mean Decrease Accuracy (MDA)” of a feature is the decrease in classification accuracy due to randomly permuting the values in that feature. For unimportant predictors, the permutation should have little to no effect on model accuracy, while permuting values of important predictors should significantly decrease it. Therefore, the greater the importance of a feature, the greater the decrease in accuracy when its values are permuted. Finally, for XGBoost, the metric “Gain” indicates the average gain across all trees that the feature is used in, which describes the relative contribution of each feature.

Feature importance was calculated by the average of over 250 replications of fivefold cross-validation. Protein analytes that were ranked in top 20 in importance measurements in all three classifiers (Table 5) were fitted as predictors in the same classifiers again. Receiver Operating Characteristic (ROC) curves and area under the curve (AUC) used to optimize feature selection were calculated using the R package caret. The data was log-transformed and auto-scaled before the ROC curves were generated. A lasso penalty is used when there are many predictors and variables that are important for prediction are selected. Since we were using variables already determined to be important, unregularized logistic regression rather than the lasso penalty was used in Fig. 8. Average AUCs were calculated with 250 repeats of fivefold cross validation, which is intended to derive a more accurate estimate of model prediction performance. Feature importances were calculated for each of the three machine learning algorithms.

## Results

### Study population characteristics

Within the study population, there were 41 females and 8 males and 40 females and 9 males in the ME/CFS and healthy controls groups respectively (Table 1). All patients who were selected met the 1994 Fukuda definition for ME/CFS. The average age and Body Mass Index (BMI) were similar between ME/CFS and control subjects and also in comparison of sexes between groups (Table 1). Seventy-nine percent of the ME/CFS patients were able to identify an acute, often flu-like, illness that immediately preceded the onset of the disease, while 20% were unaware of an initiating event and considered their onset to be gradual (Table 1), and 45 out of 49 patients had their illness for more than 3 years. The MFI-20 scores clearly depict the opposing trend of the condition of ME/CFS subjects versus controls, with a higher score reflecting the lower functional level of patients compared to the smaller score of fully functional controls (Table 1, p < 0.001). Furthermore, both the Physical and Mental Component Scores (PCS and MCS respectively) derived from the SF-36 short survey were, as expected, higher in the control group (p < 0.001, Table 1).

**Table 1:** Study population characteristics

		Controls	ME/CFS	Wilcoxon-test (p-value)	Controls	ME/CFS	Wilcoxon-test (p-value)
					(78 set)	(78 set)	
n		49	49	NA	40	38	NA
Gender	Female	41	40	NA	32	29	NA
	Male	8	9		8	9	
Age	All	51.09 ± 11.46	50.89 ± 11.40	p = 0.95	51.16 ± 11.90	51.84 ± 10.92	p = 0.79
	Female	50.94 ± 11.39	49.40 ± 11.95	p = 0.59	50.98 ± 11.93	50.08 ± 11.72	p = 0.85
	Male	51.85 ± 12.58	57.52 ± 4.82	p = 0.61	51.85 ± 12.58	57.52 ± 4.82	p = 0.61
BMI	All	25.44 ± 4.39	26.63 ± 4.87	p = 0.23	25.56 ± 4.53	27.22 ± 4.70	p = 0.08
	Female	25.35 ± 4.62	26.47 ± 5.23	p = 0.38	25.47 ± 4.86	27.17 ± 5.18	p = 0.15
	Male	25.92 ± 3.11	27.34 ± 2.86	p = 0.17	25.92 ± 3.11	27.34 ± 2.86	p = 0.17
Type of onset	Acute	NA	79.59%	NA	NA	78.95%	NA
	Gradual	NA	20.41%	NA	NA	21.05%	NA
Disease duration	< 3 years	NA	4	NA	NA	2	NA
	> 3 years	NA	45	NA	NA	36	NA
Season	Fall	48.98%	44.90%	NA	47.50%	42.11%	NA
	Summer	51.02%	55.10%	NA	52.50%	57.89%	NA
Site	Miami, FL	18.37%	20.41%	NA	17.50%	15.79%	NA
	Salt Lake City, UT	30.61%	28.57%	NA	30.00%	28.95%	NA
	Incline Village, NV	22.45%	22.45%	NA	22.50%	23.68%	NA
	New York, NY	28.57%	28.57%	NA	30.00%	31.58%	NA
MFI-20	General fatigue	7.61 ± 2.78	17.28 ± 3.70	p < 0.001	7.80 ± 2.80	17.42 ± 3.46	p < 0.001
	Physical fatigue	6.71 ± 2.45	16.57 ± 3.54	p < 0.001	6.70 ± 2.38	16.82 ± 3.50	p < 0.001
	Reduced activity	6.29 ± 2.47	15.47 ± 4.08	p < 0.001	6.25 ± 2.52	15.76 ± 3.93	p < 0.001
	Reduced motivation	6.88 ± 2.45	12.45 ± 4.17	p < 0.001	6.95 ± 2.47	12.50 ± 4.39	p < 0.001
	Mental fatigue	7.55 ± 3.29	14.8 ± 4.21	p < 0.001	7.45 ± 3.40	15.03 ± 4.28	p < 0.001
SF-36	Physical component score	55.49 ± 3.64	27.91 ± 9.68	p < 0.001	55.16 ± 3.57	27.67 ± 9.85	p < 0.001
	Mental component score	54.02 ± 7.30	40.18 ± 10.74	p < 0.001	54.13 ± 5.23	40.83 ± 10.72	p < 0.001

**Fig. 1:**
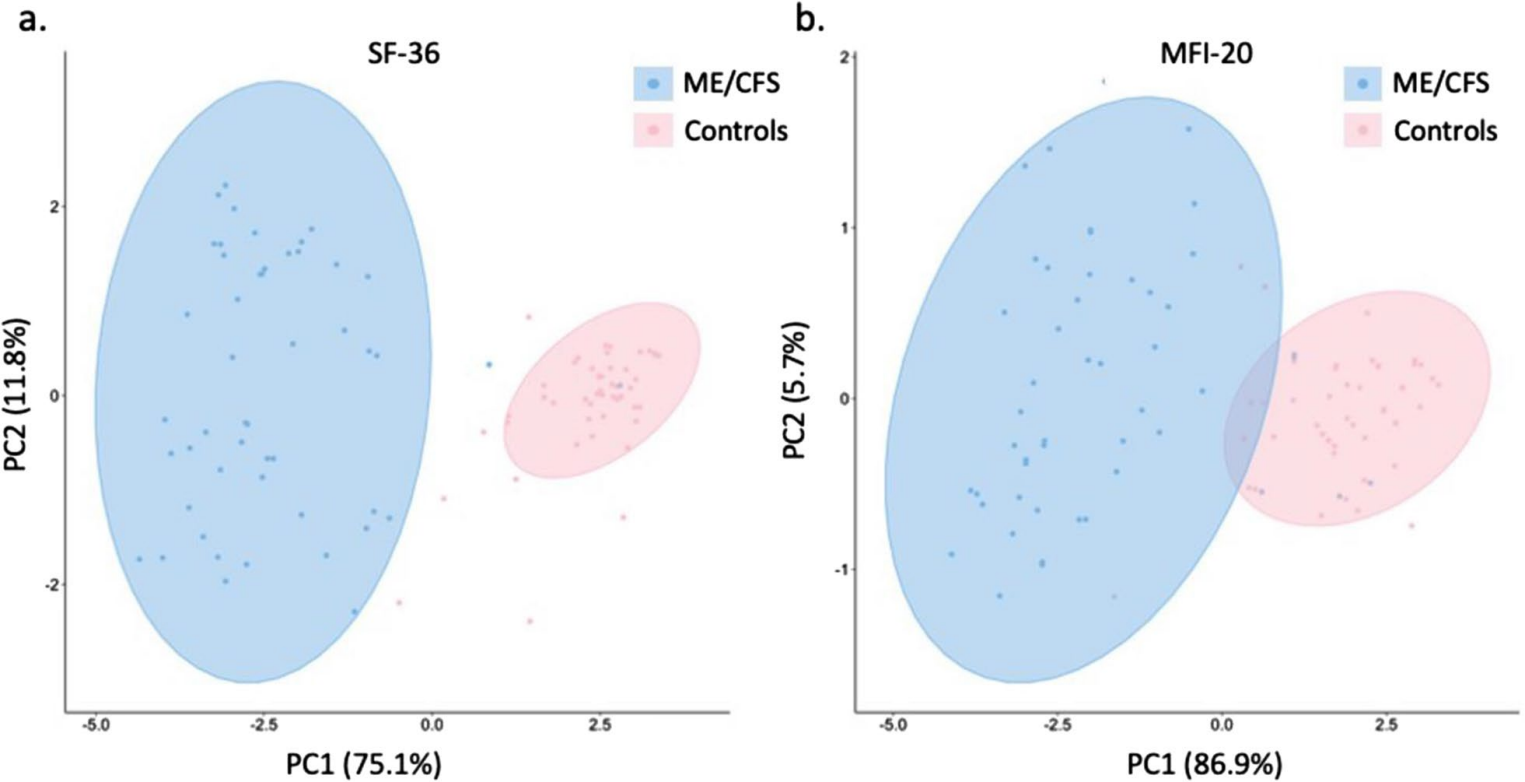
Principal Component Analysis with 95% confidence ellipses of SF-36 scores (**a**) and MFI-20 scores (**b**) in ME/CFS and controls

The Principal Component Analysis presented in Fig. 1 was performed on data obtained from the SF-36 and MFI-20 questionnaires. The first two principal components explained 86.9% (PC-1 75.1%; PC-2 11.8%, Fig. 1a) and 92.6% (PC-1 86.89%; PC-2 5.73%, Fig. 1b) of the total variance within the data set for SF-36 and MFI-20 respectively, and two significant clusters were observed, separating the ME/CFS group from the control group. Neither the season nor site where the blood was collected could distinguish groups (Additional file 1: Fig. S1).

### Size and concentrations of extracellular vesicles are different between ME/CFS and healthy controls

**Fig. 2:**
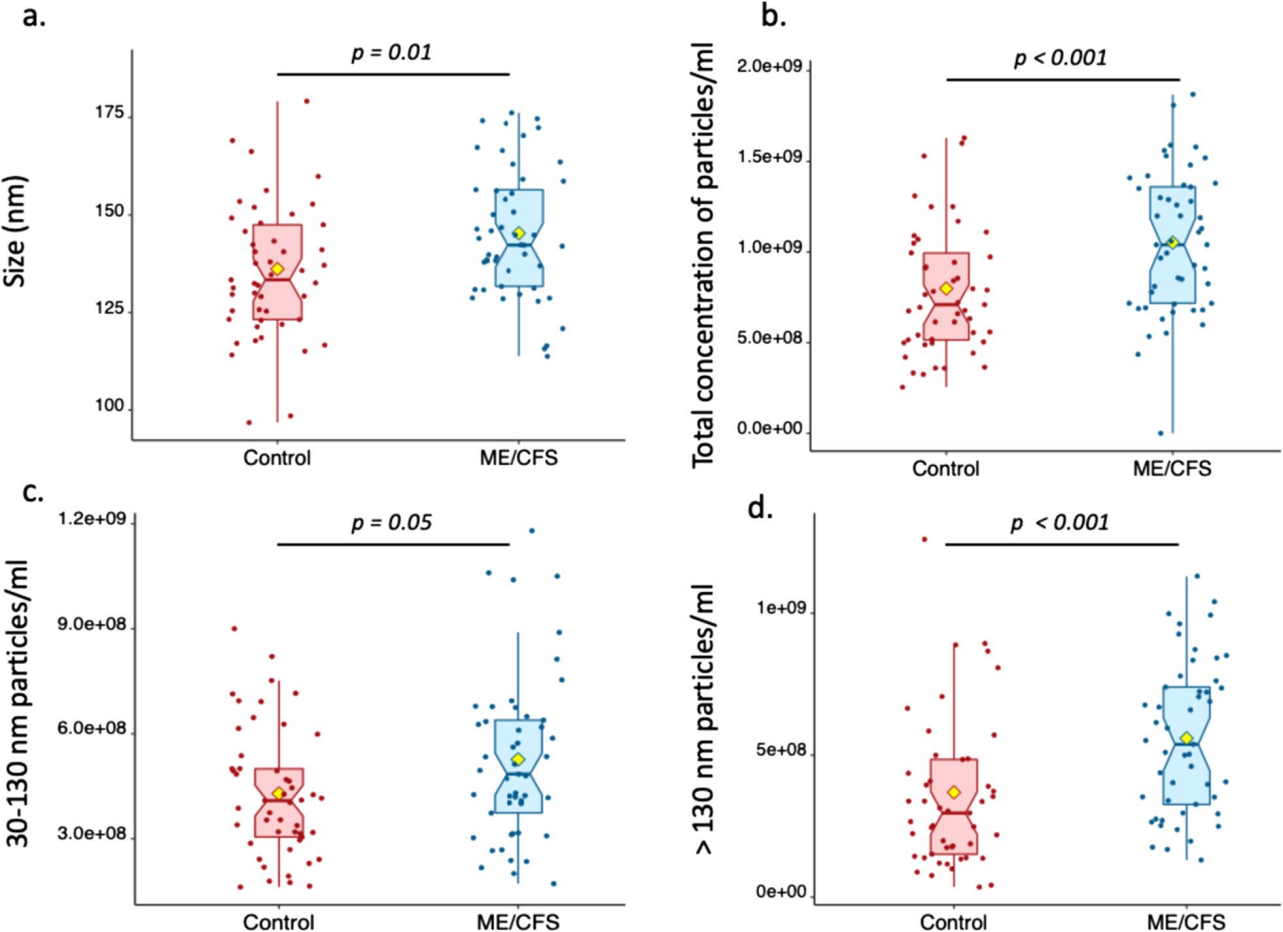
Sizing and quantification of Extracellular Vesicles. Size in nm (**a**), total concentration (**b**), 30–130 nm concentration (**c**) and > 130 nm concentration (**d**) of particles per ml of plasma in ME/CFS subjects and healthy controls as determined by Nanoparticle Tracking Analysis. The yellow diamond represents the mean

Extracellular vesicles were purified from plasma samples from ME/CFS patients and healthy individuals by precipitation and their size and concentrations analyzed by Nanoparticle Tracking Analysis (NTA) to investigate whether there were differences between clinical groups. All nanoparticles purified were smaller than 500 nm, most of them being in the typical exosome size range of 30–130 nm [40]. NTA revealed that EV particles’ size means differed between healthy individuals (136.2 ± 18.3 nm, range 97–188 nm) and ME/CFS patients (145.3 ± 16.6 nm, range 113–177 nm) (p = 0.01, Fig. 2a). The mean total concentration of particles/ml of plasma (controls: 8.0 ± 3.8 × 10^8^; ME/CFS: 10.5 ± 3.9 × 10^8^, p < 0.001, Fig. 2b), the mean concentration of EVs that ranged from 30 to 130 nm in size (controls:4.3 ± 1.8 × 10^8^, ME/CFS:5.3 ± 2.4 × 10^8^, p = 0.05, Fig. 2c) and the mean concentration of particles greater than 130 nm (controls: 3.7 ± 2.9 × 10^8^; ME/CFS: 5.6 ± 2.7 × 10^8^, p < 0.001, Fig. 2d) also exhibited a statistically significant difference between groups.

### Outlier analysis results in removal of certain subjects’ data from further consideration

We examined the number of outlier analytes across datasets. Any analyte with more than half non-detectable values was discarded, thus 6 of the 61 plasma cytokines were removed. A z-score was calculated for each subject and each analyte. Any subject/analyte pair with a two-sided q-value (p-value adjusted for FDR) less than 0.05 was considered an outlier. The resulting q-values suggested that two ME/CFS patients presented outlier profiles not initially suspected by their clinical features and therefore should be removed from the EV cytokines dataset as they represented 43% and 50% of outliers respectively (21 and 24 outliers out of 48 cytokines). For the plasma cytokines dataset, no subject had a particularly high proportion of outliers and for plasma proteomics, one ME/CFS patient presented 35% outliers (73 outliers out of 208 plasma proteins) and thus was not used in further analysis.

### Certain EV cytokine and plasma cytokine levels differ between ME/CFS and control groups

**Fig. 3:**
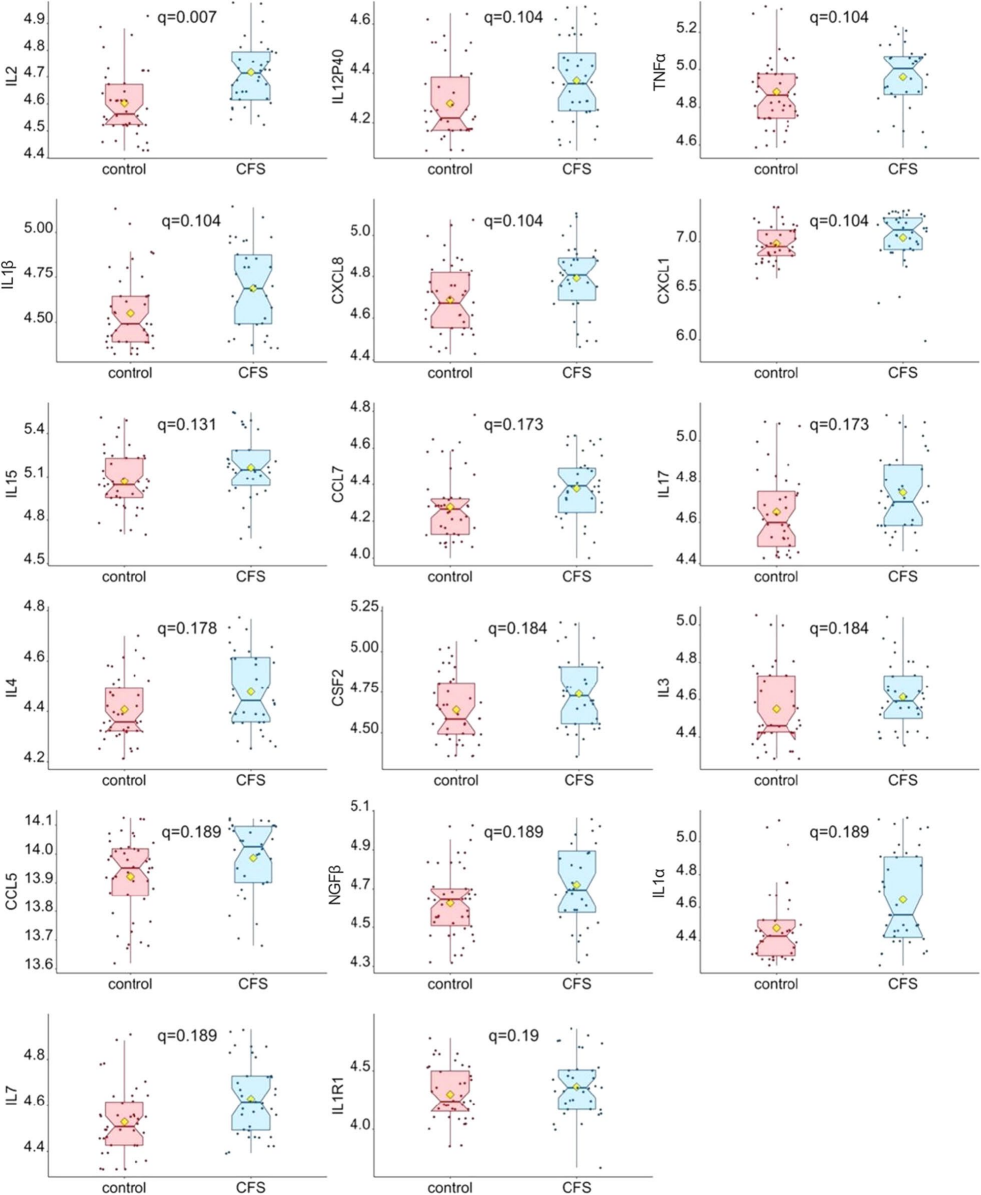
Comparison of EV log-transformed cytokine levels in ME/CFS and control subjects. Only box plots of cytokine EV levels meeting significance criteria (q < 0.2) are represented. The yellow diamond represents the mean after outliers were omitted. Wilcoxon signed-rank tests were performed, followed by Benjamini–Hochberg multiple comparison adjustment. Q-values are shown on each boxplot

We investigated differences in levels of analytes between ME/CFS patients and controls using non-parametric signed-rank Wilcoxon tests. Among the EV cytokines, levels of Interleukin 2 (IL2) were significantly different between controls and patients (q = 0.007) and the following 16 EV cytokines exhibited 0.1 < q < 0.2: IL12P40, TNFα, IL1β, CXCL8, CXCL1, IL15, CCL7, IL17, IL4, GM-CSF/CSF2, IL3, CCL5, NGFβ, IL1α, IL7, IL1R1. Figure 3 shows boxplots of the log-transformed protein levels of these 17 cytokines. For plasma cytokines [41] and plasma proteomics [14], no analyte was significantly different between cases and controls after correction for multiple comparison (FDR < 0.2). Detailed p-values, q-values, and the ratios of mean protein analyte level for the ME/CFS group versus controls can be found in the Additional file 2: Tables.

**Fig. 4:**
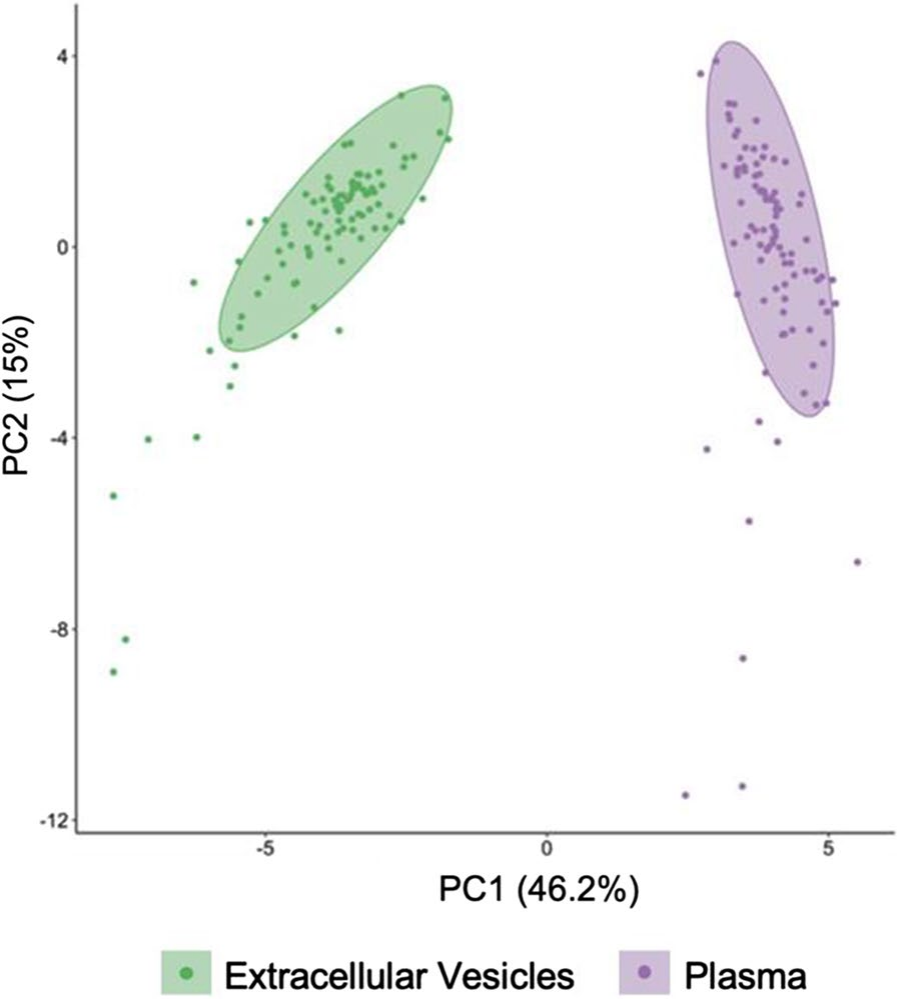
Principal Component Analysis with 95% confidence ellipses of 36 cytokine levels measured in extracellular vesicles and plasma from ME/CFS and controls

Additionally, we compared sample types within subjects with Principal Component Analysis. A total of 36 common analytes from the 48-plex EV and 55-plex plasma immunoassays were used for this analysis. The percentage of variability explained by each dimension was 46.2% for the first axis and 15% for the second axis, and two significant clusters were observed (Fig. 4).

### Numerous correlations exist within and between protein datasets

**Fig. 5:**
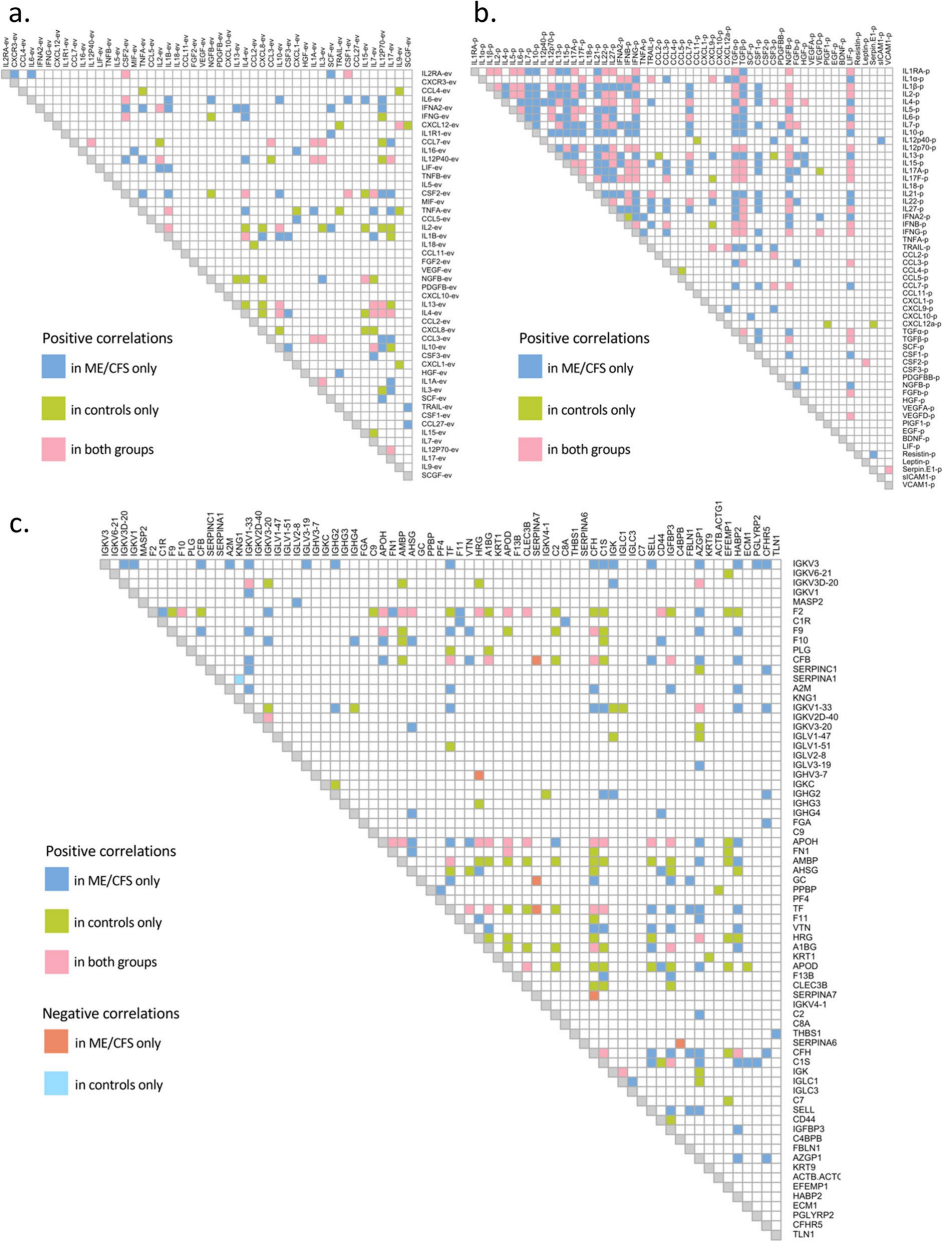
Correlograms showing correlations between EV cytokines, with coefficient |*r|*≥ 0.6 (**a**), between plasma cytokines, with |*r*|≥ 0.6 (**b**), and between plasma proteomics, with |*r*|≥ 0.8 (**c**). Filled-in boxes indicate significant correlation q < 0.01

Spearman correlation analyses were performed between datasets and are plotted as correlograms showing only significant correlations with coefficient *r* ≥ 0.6 (Fig. 5). A total of 316 positive significant correlations were found in ME/CFS subjects and 300 in controls between cytokine levels in EV samples (q < 0.01) and 88 and 73 had strong Spearman correlation coefficients (r ≥ 0.6) in the ME/CFS and control groups, respectively (Fig. 5a). Thirty-four of them were common to both groups (pink squares, Fig. 5a). When correlating plasma cytokines to each other, the ME/CFS cohort had 710 significant correlations including 327 at r ≥ 0.6 (q < 0.01), and the control group had 394 with 146 at r ≥ 0.6 (q < 0.01); 136 were common to both groups (Fig. 5b). In both EV and plasma cytokine correlation analysis, no significant negative correlations were found, and there was a higher number of positive correlations in the ME/CFS cohort as compared to the healthy individuals (Fig. 5a, b).

We also investigated correlations between the 55 plasma and 48 EV cytokine levels (Additional file 1: Fig. S2). No negative and few positive significant correlations were found in both groups (15 and 13 for ME/CFS and controls respectively at r ≥ 0.5, with 4 common to both groups). Amongst these significant correlations, levels of LIF in EVs correlated with 8 plasma cytokines in ME/CFS (CCL3, IL15, LIF, IL17, IL21, IFNβ, TGFα and TGFβ) and 5 in the control group (CCL3, IL1α, IL17, IL21 and IFNβ) (Supplemental Fig. 2).

For plasma proteomics, 160 and 130 significant positive correlations were found in the ME/CFS and control groups, respectively, with a Spearman coefficient r greater than 0.8 (q < 0.01) (Fig. 5c) and 42 were common to both groups (pink squares, Fig. 5c). Six pairs of proteins were significantly and negatively correlated in the ME/CFS group only (orange squares, Fig. 5c), with 3 including SERPINA7, and one unique to the control group (SERPINA1/KNG1, r = − 0.82, q < 0.01, light blue square, Fig. 5c).

When analyzing relationships between the plasma proteomics dataset with either the EV cytokines or the plasma cytokines datasets, only one significant correlation was found between an EV protein and a protein assayed by mass spectrometry in the control group (CXCL12-ev/PROZ, r = 0.69, q = 0.014).

### Correlations of protein levels with clinical metadata indicate their importance in disease state

All proteins were analyzed for correlations with the clinical metadata using the same methods previously described. Only significant results after adjustment for multiple comparison (q < 0.1) are shown in Table 2. There were significant correlations between plasma cytokines, plasma proteomics and the clinical metadata, but none were found with the EV cytokine dataset (Table 2).

**Table 2:** Correlations between clinical data and plasma cytokines (top) and plasma proteomics (bottom)

Plasma cytokines	Cohort	Metadata	Correlation	P-value	Q-value
CSF2	ME/CFS	Sex (0 = female, 1 = male)	− 0.474	0.001	0.032
	Control	Sex (0 = female, 1 = male)	− 0.465	0.001	0.042
	ME/CFS	IBS (0 = without IBS, 1 = with IBS)	0.437	0.002	0.052
	ME/CFS	BMI	0.596	0.000	0.000
	Control	BMI	0.448	0.001	0.023
	ME/CFS	Physical function (SF-36)	− 0.539	< 0.001	0.002
	ME/CFS	Physical component summary (SF-36)	− 0.459	0.001	0.035
	ME/CFS	General Fatigue (MFI-20)	0.436	0.002	0.047
Leptin	ME/CFS	Sex (0 = female, 1 = male)	− 0.414	0.003	0.086
	Control	Sex (0 = female, 1 = male)	− 0.427	0.002	0.061
	ME/CFS	IBS (0 = without IBS, 1 = with IBS)	0.433	0.002	0.052
	ME/CFS	BMI	0.577	< 0.001	< 0.001
	Control	BMI	0.475	0.001	0.018
	ME/CFS	Physical function (SF-36)	− 0.558	< 0.001	0.002
	ME/CFS	Physical component summary (SF-36)	− 0.445	0.001	0.035
	ME/CFS	General fatigue (MFI-20)	0.439	0.002	0.047
CCL2	ME/CFS	Age	0.440	0.002	0.060
CXCL10	ME/CFS	Age	0.394	0.005	0.099
CCL11	ME/CFS	Age	0.431	0.002	0.060
TNFα	ME/CFS	BMI	0.543	< 0.001	0.001
	ME/CFS	Physical function (SF-36)	− 0.508	< 0.001	0.004
	ME/CFS	Physical component summary (SF-36)	− 0.432	0.002	0.035
IL1RA	ME/CFS	BMI	0.468	0.001	0.010
	ME/CFS	Physical function (SF-36)	− 0.480	< 0.001	0.007
IL13	ME/CFS	Reduced activity (MFI-20)	0.482	< 0.001	0.025

Plasma proteomics	Cohort	Metadata	Correlation	P-value	Q-value
PROS1	Control	Physical component summary (SF-36)	− 0.608	< 0.001	0.008
	Control	General fatigue (MFI-20)	0.590	< 0.001	0.016
	Control	Vitality (SF-36)	− 0.590	< 0.001	0.015
FCRL3	Control	Vitality (SF-36)	− 0.538	< 0.001	0.039
IGHV3-23.IGHV3-30	Control	Vitality (SF-36)	0.527	< 0.001	0.039
SERPINA5	ME/CFS	General health (SF-36)	0.646	< 0.001	0.004
	ME/CFS	Social functioning (SF-36)	0.593	< 0.001	0.027
CETP	Control	General fatigue (MFI-20)	0.547	< 0.001	0.032
	Control	Total (MFI-20)	0.557	< 0.001	0.025
HBA1	Control	Mental fatigue (MFI-20)	− 0.558	< 0.001	0.046
	Control	Total (MFI-20)	− 0.556	< 0.001	0.025

**Fig. 6:**
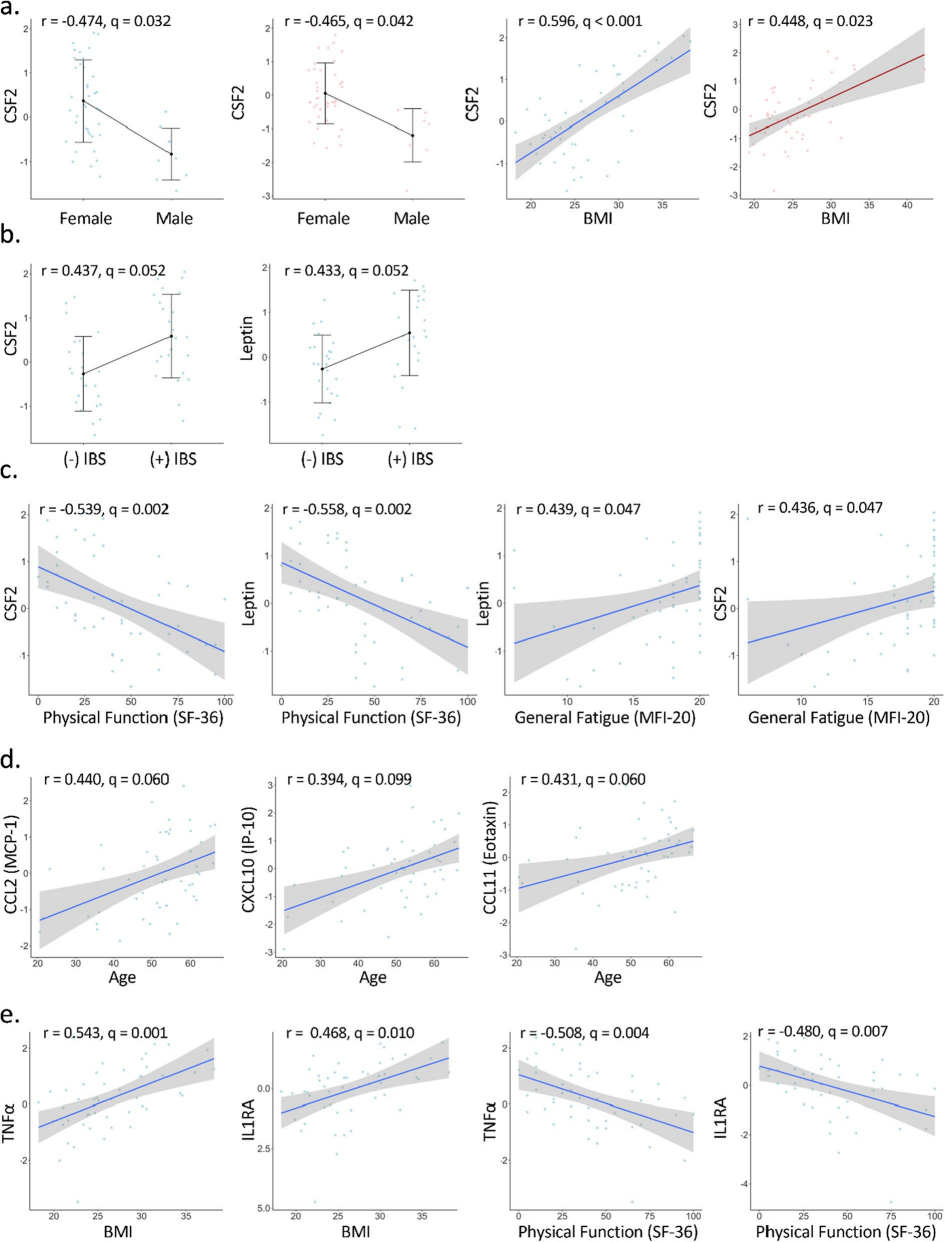
Significant plasma cytokines correlation plots with metadata. Blue dots and lines are for ME/CFS subjects and pink dots and lines for controls

Within the plasma cytokine dataset, both Colony Stimulating Factor 2 (CSF2) and leptin were negatively correlated with sex and positively correlated with BMI in both the ME/CFS and control groups (Fig. 6a). Interestingly, individuals with ME/CFS and IBS have higher concentrations of CSF2 and leptin than people with ME/CFS and without IBS, and these correlations were not observed in the control group (Fig. 6b).

The ME/CFS cohort also revealed unique significant correlations with the health questionnaire data related to physical function (SF-36) and fatigue (MFI-20) that were not found in the control group. CSF2 and leptin were negatively correlated with Physical Function (r = − 0.539, q = 0.002 for CSF2; r = − 0.558, q = 0.002 for leptin) and the Physical Component Summary (r = − 0.459, q = 0.035 for CSF2; r = − 0.445, q = 0.035 for leptin), and positively correlated with General Fatigue (r = 0.439, q = 0.047 for CSF2; r = 0.436, q = 0.047 for leptin) (Table 2, Fig. 6c).

We found other significant correlations between cytokines and the clinical data in ME/CFS subjects that were not found in controls: CCL2, CXCL10, and CCL11 were positively correlated with age (r = 0.440, q = 0.060 for CCL2; r = 0.394, q = 0.099 for CXCL10; r = 0.431, q = 0.060 for CCL2) (Fig. 6d). Both (TNFα and IL1RA were positively correlated with BMI (r = 0.543, q = 0.001 and r = 0.468, q = 0.010 respectively), and negatively correlated with the Physical Function category of the SF-36 (r = − 0.508, q = 0.004 and r = − 0.480, q = 0.007 for TNFα and IL1RA respectively) (Fig. 6e). Lastly, IL13 positively correlated with the Reduced Activity score from the MFI-20 questionnaire (r = 0.482, q = 0.025).

**Fig. 7:**
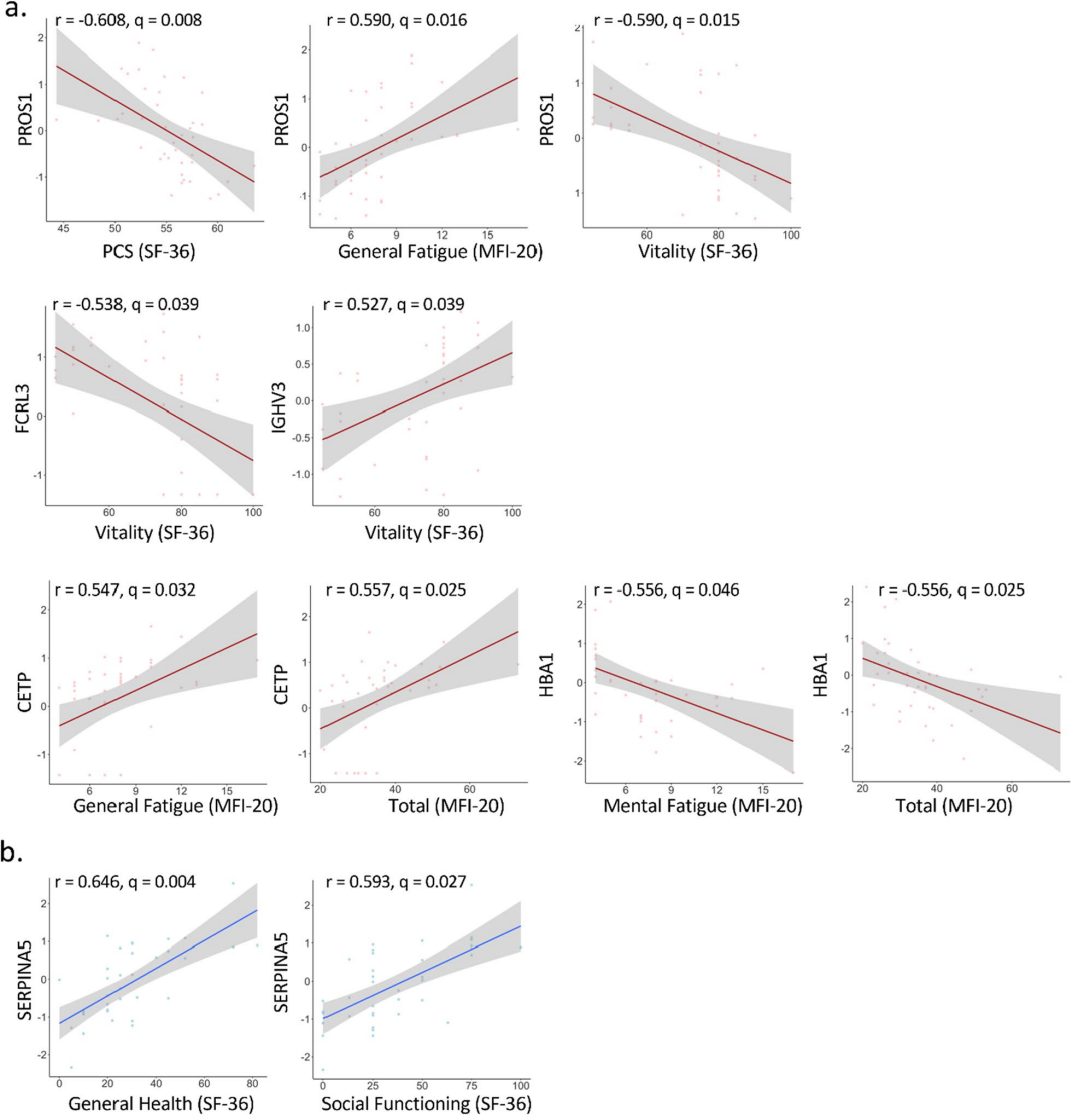
Significant plasma proteomics correlation plots with metadata in controls (**a**) and in ME/CFs (**b**)

As mentioned above, additional significant correlations were found between the plasma proteomics dataset and the clinical metadata. There were 9 significant correlations in the control group and only two in the ME/CFS subjects (Table 2, bottom part). In control samples, Protein S (PROS1) and Fc Receptor Like 3 (FCRL3) were negatively correlated with Vitality (r = − 0.590, q = 0.015 for PROS1, r = − 0.538, q = 0.039 for FCRL3). Additionally, PROS1 was negatively correlated with the SF-36 Physical Component Summary (r = − 0.608, q = 0.008) and positively correlated with the MFI-20 General Fatigue score (r = 0.590, q = 0.016). The Cholesteryl Ester Transfer Protein (CETP) was positively correlated with General Fatigue (r = 0.547, q = 0.032) and Total scores from the MFI-20 (r = 0.557, q = 0.025), and the Hemoglobin Subunit Alpha 1 (HBA1) was negatively correlated with the two same scores (r = − 0.558, q = 0.046 and r = − 0.556, q = 0.025 respectively) (Fig. 7a). In the ME/CFS group, Serpin Family A Member 5 (SERPINA5) was positively correlated with General Health (r = 0.646, q = 0.004) and Social Functioning (r = 0.593, q = 0.027) from the SF-36 questionnaire (Fig. 7b).

### Robust linear regression reveals additional relationships between certain proteins and clinical information

In order to better understand the relationship between proteins and the metadata, we performed robust linear regression and t-tests for the estimated coefficients. Robust linear regression was performed for EV cytokines, plasma cytokines, and plasma proteomics, respectively. Each model included a specific protein level as the predicted variable and the cohort (ME/CFS or control), sex, age, BMI, and Irritable Bowel Syndrome (IBS) as a covariate. Interactions between cohort and the metadata covariates were also included in the model. The interactions test the hypothesis that the relationship between the metadata and the level of a protein is different in ME/CFS than in the control group. The significant effects are summarized in Table 3. It is standard practice in biostatistics to include both main effects whenever two variables have a statistically significant interaction. The reasoning here is that the interaction shows that the variables are having effects even if the main effect does not achieve statistical significance. We followed this practice. In Table 3, Male is a dummy (indicator or 0–1) variable that equals 1 for males and 0 for females. Similarly, ME/CFS is a dummy variable that is 1 or 0 for cases or controls, respectively, and IBS is a dummy variable equal to 1 or 0 for subjects with or without IBS, respectively. ME/CFS:Age is the product of ME/CFS and Age and so is equal to 1for ME/CFS cases and equal to 0 for controls. ME/CFS:Male is the product of two dummy variables and so is equal to 1 for males in the ME/CFS group and equal to 0 for all other subjects. ME/CFS:( +) IBS is equal to 1 for ME/CFS cases with IBS and 0 otherwise.

**Table 3:** Effects of clinical data on EV, plasma cytokines and plasma proteomics according to robust linear regression

EV cytokines	Covariate-Interaction^a^	β coefficient	P-value	Q-value
CXCL1	Age	− 0.013	0.001	0.035
CCL11	Age	0.032	0.001	0.035

Plasma cytokines	Covariate-Interaction^a^	β coefficient	P-value	Q-value
Leptin	BMI	0.123	< 0.001	< 0.001
	Male	− 1.119	< 0.001	0.001
CSF2	BMI	0.123	< 0.001	< 0.001
	Male	− 1.230	< 0.001	< 0.001
CCL2	Intercept	0.690	0.486	0.900
	ME/CFS	− 2.696	0.046	0.886
	Age	− 0.037	0.006	0.152
	ME/CFS:Age	0.075	< 0.001	0.009
CSF3	Intercept	0.576	0.573	0.900
	ME/CFS	− 1.994	0.151	0.886
	Age	− 0.047	0.001	0.038
	ME/CFS:Age	0.075	0.001	0.009

Plasma proteomics	Covariate-Interaction^a^	β coefficient	P-value	Q-value
SAA1	Age	0.047	< 0.001	0.049
PFN1	Intercept	0.809	0.322	0.996
	ME/CFS	− 0.996	0.417	0.914
	Male	0.081	0.795	0.967
	ME/CFS:Male	− 1.901	< 0.001	0.030
IGHA2	Intercept	0.454	0.561	0.996
	ME/CFS	− 2.048	0.084	0.907
	( +) IBS	− 2.811	0.001	0.137
	ME/CFS:( +) IBS	3.467	< 0.001	0.019
LRG1	Intercept	0.261	0.727	0.996
	ME/CFS	− 1.082	0.336	0.907
	( +) IBS	− 2.502	0.001	0.153
	ME/CFS:( +) IBS	3.093	< 0.001	0.026

Similarly, ME/CFS:Age or ME/CFS:( +) IBS indicate the interaction term between ME/CFS and age or subjects with IBS. ( +) IBS denotes subjects with IBS. Categorical variables were coded as follows: Cohort: control = 0; ME/CFS = 1; IBS: no IBS = 0; with IBS = 1

^a^ME/CFS:Male indicates the interaction term between ME/CFS and males

In EV cytokine samples, age was significant for predicting CXCL1 level (β = − 0.013, q = 0.035) and CCL11 level (β = 0.032, q = 0.035). Thus, CXCL1 decreases with age but CCL11 increase with age. (Table 3).

In plasma cytokines, both BMI and Male significantly predicted Leptin and CSF2 levels. The effect for the dummy variable Male is the difference between the means for males and female. For example, the mean of the variable Leptin (or CSF2) is, all else equal, 1.119 (or 1.230) lower for males compared to females. For both CCL2 and CSF3, the main effect of age, and the interaction term between age and cohort were significant. The intercepts for the regression of CCL2 on age are 0.690 and 0.690− 2.696 = − 2.006 for controls and cases, respectively. For every one-year increase in age, the average of CCL2 will decrease by 0.037 in controls and increase by 0.075–0.037 = 0.038 in cases. The intercepts for the regression of CSF3 on age are 0.576 and 0.576−1.994 = − 1.418 for controls and cases, respectively. For every one-year increase in age, the average of CFS3 will decrease by 0.047 in controls and increase by 0.075–0.047 = 0.028 in cases.

In plasma proteomics data, age was also significant for predicting SAA1 level (β = 0.047, q = 0.049). For PFN1, the interaction between ME/CFS and sex was significant (β = − 1.901, q = 0.030). The mean of PFN1 is 0.809 for female controls, 0.809−0.996 = − 0.187 for female cases, 0.809 + 0.081 = 0.890 for male controls, and 0.809 + 0.081−0.996−1.901 = − 2.007 for males cases. Thus, mean PFN1 is higher in controls than in cases for both sexes, but the difference is much greater in males (0.809 + 0.187 = 0.996 for females versus 0.890 + 2.007 = 2.897 for males).

For IGHA2, the interaction between ME/CFS and IBS was significant (β = 3.467, q < 0.001). The mean of IGHA2 is 0.454 for controls without IBS, 0.454−2.048 = − 1.594 for ME/CFS cases without IBS, 0.454−2.811 = − 2.357 for controls with IBS, and 0.454−2.048−2.811 + 3.467 = − 0.938 for cases with IBS. Therefore, mean IGHA2 is higher for controls than cases for subjects without IBS but higher in cases than controls for subjects with IBS. For LRG1, the interaction between ME/CFS and IBS was significant (β = 3.093, q < 0.001). The mean of LRG1 is 0.261 for controls without IBS, 0.261−1.082 = − 0.821 for ME/CFS cases without IBS, 0.261−2.502 = − 2.241 for controls with IBS, and 0.261−1.082−2.502 + 3.093 = 0.230 for cases with IBS. We see that mean LRG1 is higher in controls than cases for subjects without IBS but higher in cases than controls for subjects with IBS (Table 4).

**Table 4:** Means of the log-transformed protein levels PFN1, IGHA2, and LRG1 for subpopulations defined by pairs of binary variables

Proteins		Controls	ME/CFS
PFN1	Female	0.809	− 0.187
	Male	0.890	− 2.007
IGHA2	(− ) IBS	0.454	− 1.594
	( +) IBS	− 2.357	− 0.938
LRG1	(− ) IBS	0.261	− 0.821
	( +) IBS	− 2.241	0.230

( +) or (− ) IBS denote subjects with or without IBS respectively

IBS has opposite effects in cases and controls on IGHA2 and LRG1 (Table 4). Although these differences are statistically significant, it should be noted that there was only one control subject with IBS.

### Three machine learning approaches result in predictive and discriminative models

**Fig. 8:**
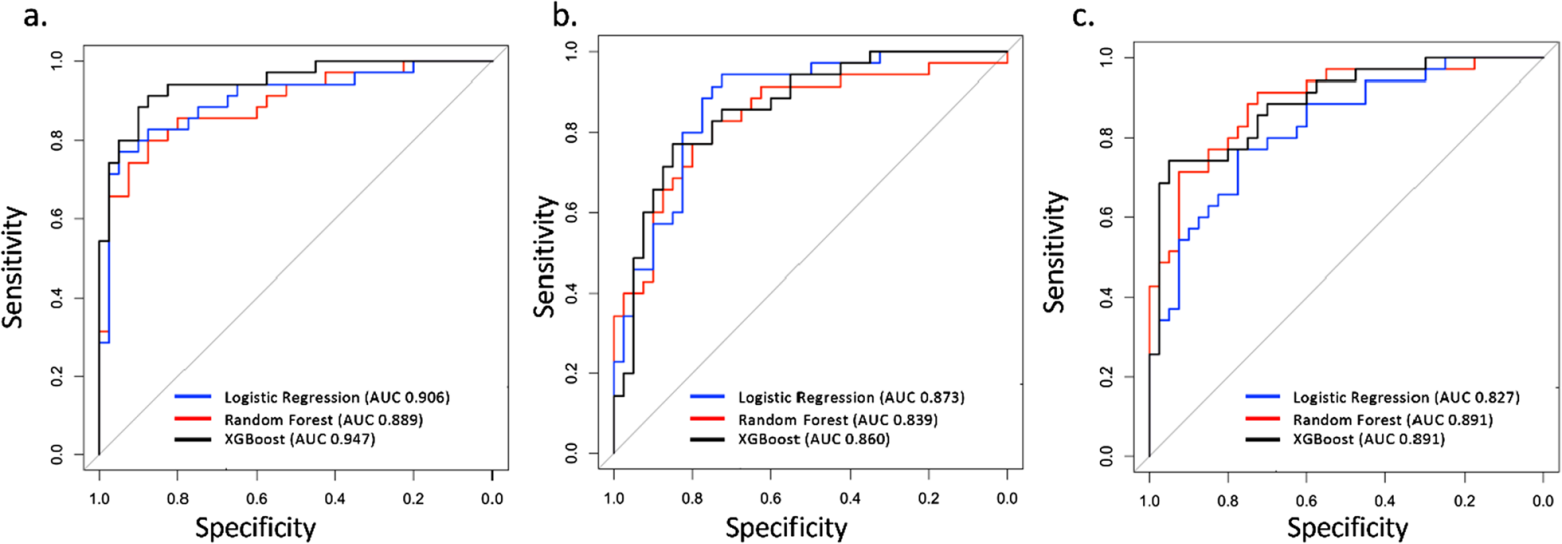
Predictive performance (AUROC) for distinguishing ME/CFS from controls with the top 20 (**a**), top 8 (**b**) and the 7 protein analytes common to all three classifiers (**c**). Unregularized logistic regression rather than the lasso penalty was used in this figure, since we were using variables known to be important

The top 20 protein analytes and feature importance values for each of the three machine learning approaches can be found in Table 5. All three methods had an excellent performance at distinguishing ME/CFS from controls using the top 20 protein analytes with 250 replications of fivefold cross-validation. Figure 8 shows the ROC curves and the AUROC values from these three classifiers with the top 20 proteins ranked in importance measurements. The XGBoost classifier performed the best with a high degree of accuracy (86.1%, Additional file 1: Fig. S3a) with a cross-validated AUROC value of 0.947 (95% CI 0.895–0.998). Furthermore, using the top 8 proteins from each classifier, logistic regression (LASSO) gave the best results with an AUC of 0.873 (95% CI 0.792–0.953) and accuracy of 78.6% (Fig. 8b and Additional file 1: Fig. S3b). Finally, Random Forest with 7 protein analytes common to all three top 20 lists (bold proteins in Table 5) distinguished ME/CFS from the controls with an AUROC value of 0.891 (95% CI 0.817–0.966) and accuracy of 79.1% (Fig. 8c and Additional file 1: Fig. S3c).

**Table 5:** Top 20 proteins ranked in importance measurements in LASSO, Random Forest and XGBoost

Logistic Regression with LASSO penalty			Random Forest			XGBoost		
Protein Name	Direction	Percentage	Protein Name	Direction	MDA	Protein Name	Direction	Gain
**IL2-ev**	Increased	46.4	**CRTAC1**	Decreased	2.939	**CRTAC1**	Decreased	0.044
**CAMP**	Increased	43.2	CCL5-ev	Increased	2.759	CCL5-ev	Increased	0.033
IGHA1	Decreased	40	**IGF1**	Decreased	2.509	**CAMP**	Increased	0.033
**IGLV1-47**	Decreased	36	NGFB-ev	Increased	2.423	**IGF1**	Decreased	0.022
**CRTAC1**	Decreased	36	CXCL8-ev	Increased	2.394	**IGLV1-47**	Decreased	0.021
LRG1	Decreased	30.8	**IL2-ev**	Increased	2.377	**IL2-ev**	Increased	0.020
IGF1	Decreased	28.8	CPB2	Increased	2.325	LRG1	Decreased	0.019
PVR	Decreased	24.8	IL12p70-p	Decreased	2.14	CXCL8-ev	Increased	0.018
KNG1	Increased	22.8	**IGLV1-47**	Decreased	1.897	IL22-p	Decreased	0.015
**TUBA1B/A/C**	Decreased	7.6	IGLV3-10	Decreased	1.828	TNFα-p	Increased	0.013
IGFALS	Decreased	6	**IGFALS**	Decreased	1.794	IGFALS	Decreased	0.012
VEGF-ev	Increased	5.2	**CAMP**	Increased	1.758	IGHA1	Decreased	0.012
IGLV2-11	Decreased	5.2	CCL7-ev	Increased	1.593	GSN	Decreased	0.011
C4BPB	Increased	2.4	LRG1	Decreased	1.486	**TUBA1B/A/C**	Decreased	0.011
BTD	Decreased	1.2	HBB	Increased	1.366	CXCL10-ev	Decreased	0.011
IL7-p	Decreased	0.4	IL15-ev	Increased	1.337	HBB	Increased	0.010
TNFα-p	Increased	0.4	**TUBA1B/A/C**	Decreased	1.196	MASP1	Decreased	0.009
IGKV1D-16	Increased	0.4	LCAT	Decreased	1.048	HYI	Decreased	0.009
APOC1	Increased	0.4	IGFBP3	Decreased	1.024	CCL7-ev	Increased	0.009
MBL2	Increased	0.4	PKM	Decreased	1.021	IGLV3-10	Decreased	0.009

Direction is measured relative to controls. Proteins followed by “p” or “ev” come from the plasma and EV cytokines datasets respectively, all other proteins are from the plasma proteomic dataset

## Discussion

In this study, we utilized samples and data from 98 of the 100 subjects who previously provided samples that were analyzed for fecal metagenomics and plasma cytokines [26] and also for plasma proteins assayed by mass spectrometry [14]. Furthermore, extracellular vesicles were isolated from these 98 samples, and we found that the mean size and concentrations of particles were significantly higher in ME/CFS (Fig. 2). Although a previous report using the same EV purification method as the present study found that the mean size of ME/CFS EVs was reduced [42], the authors analyzed EVs isolated from 10 ME/CFS patients and 5 healthy controls vs. 49 ME/CFS and 49 controls in this study, did not use thrombin to remove fibrinogen and used low centrifugal forces to pellet EVs (1500 g vs. 12,000 g in this study). All together this could explain the different results observed with our current study. Finally, our results confirmed other findings reporting higher concentration of vesicles in ME/CFS [24, 25, 42] and these observations are also seen in conditions such as Alzheimer’s disease [17] and cerebrovascular disease [20].

**Fig. 9:**
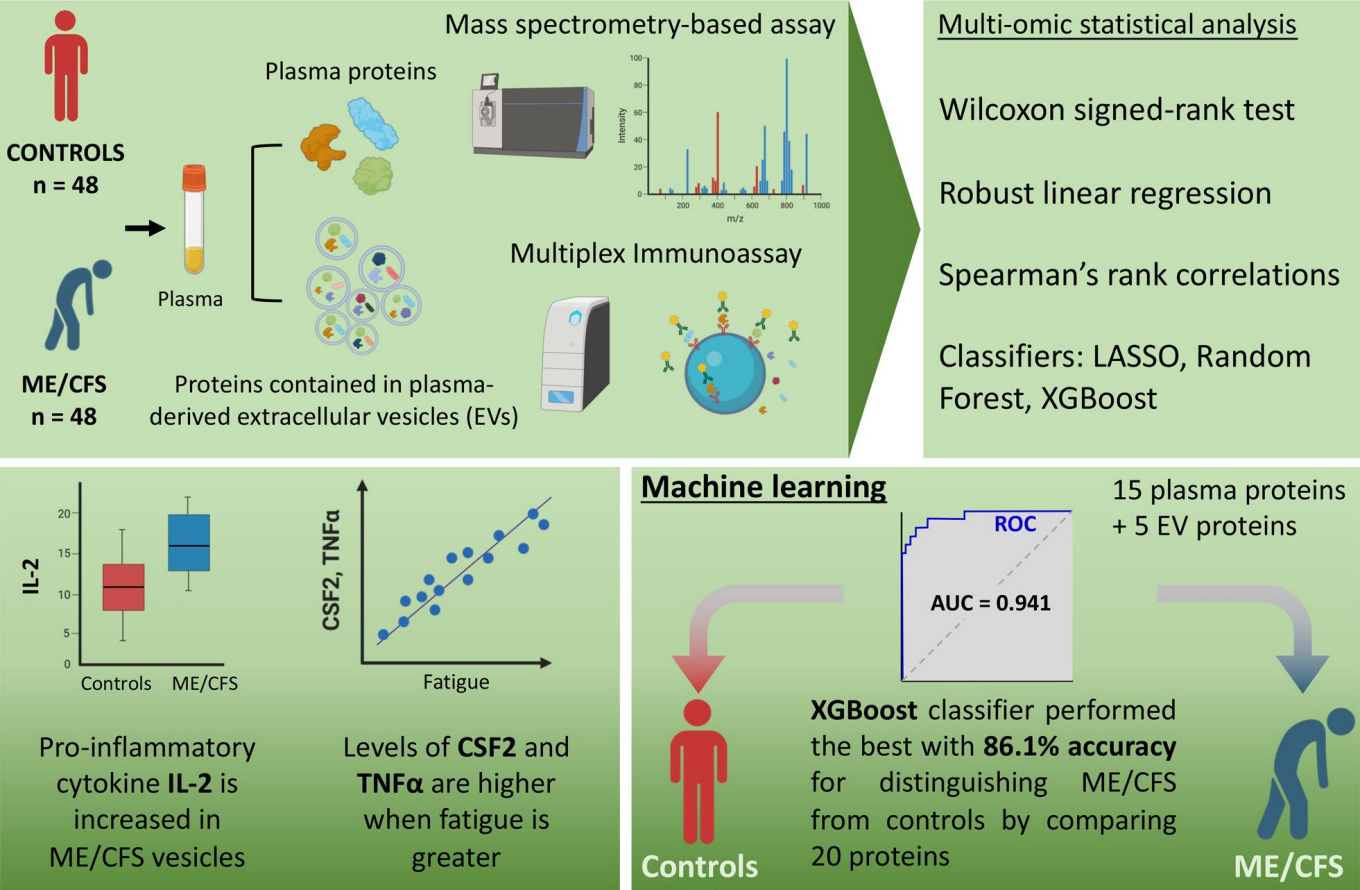
Graphical summary of key findings from this study

Our work demonstrates the value of using multiple assays on the same samples, and also the importance of performing correlations with clinical data. Doing so has allowed us to identify a number of associations of particular proteins with patient symptoms. Importantly, we demonstrate that the data can distinguish between patients and controls at high accuracy. ME/CFS has long been incorrectly viewed by some as a psychological illness. Being able to separate patients and controls through analyses of plasma is a strong demonstration of the biological natures of the illness. A summary of our experimental assays and key findings is shown in Fig. 9.

Our current analysis of 98 samples agrees with the prior comparison of plasma cytokines in 100 ME/CFS and controls, which did not identify any significant differences between cohorts after adjustment for multiple testing [26]. In contrast, we identified 17 EV cytokines that distinguish patients and controls with adjusted p-values of less than 0.2, all higher in ME/CFS subjects. Out of these 17 proteins, the majority (10 out of 17) are known to be pro-inflammatory cytokines/chemokines (TNFα, IL1β, CXCL8, CXCL1, IL15, CCL7, IL17, CCL5, IL1α and IL1R1), 5 are related to adaptive immunity (IL2, CSF2, IL3, IL4 and IL7), IL12p40 has anti-inflammatory properties and NGFβ is both pro- and anti-inflammatory. Higher levels of pro-inflammatory cytokines are in line with previous reports [43–45].

Although differences in EV cytokine levels did not reach statistical significance after correction for multiple comparison in a prior pilot study with only 38 subjects, 13 of the 17 EV cytokines in the present study were also found at higher levels in EVs from ME/CFS subjects in comparison to controls [25]. The most significant difference was IL2 (q = 0.007, Fig. 3). IL2 is a secreted cytokine produced by activated CD4 + and CD8 + T lymphocytes and promotes strong proliferation of activated B-cells and subsequently immunoglobulin production. It plays a pivotal role in regulating the adaptive immune system by controlling the survival and proliferation of regulatory T-cells. IL2 levels were found to be higher in cerebrospinal fluid [46] and plasma from ME/CFS patients [47]. The higher levels of IL2 found in EVs in the present study might be part of a specific immune response in ME/CFS. A number of cytokines/chemokines which were observed to be dysregulated are either produced by B cells or are also B cell regulators (e.g. CXCL1 and CXCL12).

Correlations of cytokines with other cytokines provide information about the networks of interactions between signaling molecules. Several other studies demonstrated that the networks of plasma or extracellular vesicle cytokines differ between ME/CFS subjects and controls [25, 41, 44, 48]. We have chosen to display correlations between the three types of data: plasma and EV cytokines and plasma proteomics—using correlograms. Inspection of the visual representation of these protein–protein interactions immediately reveals that there are positive correlations between EV cytokines and between plasma cytokines that occur in cases but not controls and vice versa (Fig. 5). A particularly striking observation is a greater number of positive correlations between plasma cytokines in ME/CFS than in controls (Fig. 5b), indicating that cytokine signaling is substantially different, perhaps reflective of an inflammatory environment.

Seventy-one proteins characterized by mass spectrometry exhibited significant correlations with other plasma proteins (Fig. 5c). For example, F2 exhibited 31 positive correlations with other proteins, of which eleven were seen in cases but not controls. F2 is coagulation factor II or thrombin, and converts fibrinogen to fibrin and activates factors V, VII, VIII, XIII. Thrombin promotes platelet activation and aggregation, but it is also thought to have other functions during inflammation and wound healing [49].

Despite not observing significant differences in levels of plasma cytokines between the two cohorts, we did observe correlations of plasma cytokines with clinical data. CSF2, also known as Granulocyte Monocyte Colony Stimulation factor (GM-CSF), is lower in males in both ME/CFS and controls and increases with BMI in both cohorts according to both the robust linear regression and correlation analyses (Fig. 6a, Tables 2 and 3). In the ME/CFS cohort, with increasing CSF2, scores on the SF36 Physical Function and the MFI-fatigue scales indicate greater impact of physical and fatigue symptoms, respectively. Increase in GM-CSF is associated with chronic inflammation [50]. GM-CSF induces classical monocytes to differentiate into monocyte-derived dendritic cells and macrophages in vitro [51]. Classical monocytes exhibit a unique gene expression pattern in ME/CFS compared to controls [52], and elevated GM-CSF could be a signaling factor involved in this response.

Increases in levels of three cytokines, CCL2, CXCL10, and CCL11 were associated with increasing age only in the ME/CFS cohort, according to Spearman correlations. CCL2, also known as MCP-1 (Monocyte Chemoattractant Protein-1), attracts monocytes across the endothelium into tissues [53], and could also be a factor in the altered monocyte gene expression profile [54]. CCL2 was also observed to decrease with age in the total cohort by both robust linear regression and Spearman correlation, but increases in the ME/CFS cohort with increasing age, according to Spearman correlation (Fig. 6d, Table 3). Using robust linear regression, plasma CCL11 was not significantly increasing with age but EV CCL11 was predicted to be higher with increasing age. CXCL10 (IP10) is also involved in cell migration, in particular, attraction of macrophages, monocytes and activated T and NK cells [55]. CCL11, also known as eotaxin, is known to increase with aging and higher levels are associated with decreased neurogenesis [56]. Two large studies previously observed an association of leptin, GM-CSF, IP10, and eotaxin with ME/CFS severity [43] or higher eotaxin in long-term ME/CFS cases [41]. Almost all of the ME/CFS subjects in this study have been ill more than 3 years.

Higher leptin is correlated with female sex and higher BMI in both patients and controls both by robust linear regression and correlation analyses (Tables 2 and 3). Higher leptin is also associated with IBS in the patient cohort (Fig. 6b). Increase in leptin is also correlated with worse scores on the SF36 physical function measures and MFI-fatigue scale (Fig. 6c). Leptin was previously correlated with fatigue and severity in ME/CFS [43, 54]. Increasing levels of another inflammatory cytokine, TNFα, also correlates with lower patient Physical Function scores on the SF36 and has previously been reported to be elevated in ME/CFS [41, 57, 58] (Fig. 6e).

Higher levels of IL1-RA, which antagonizes IL1 inflammatory cytokines, were associated with higher BMI and lower SF-36 Physical Function in ME/CFS cases (Fig. 6e). Although IL1-RA could be considered to be anti-inflammatory, it is known that IL1-RA levels are higher in obesity [59] and higher levels are considered to be a marker for metabolic dysregulation [60], which could be resulting in the lower physical ability.

We observed that lower levels of the anti-inflammatory cytokine IL13 were associated with lower activity in the ME/CFS cases. IL-13 was previously reported to be lower in females with ME/CFS vs. controls [61]. In contrast, higher IL13 was correlated with increased symptom severity in one study [43], while no difference between cases and controls was seen in another [41].

Higher levels of another protein associated with hemostasis, PROS1, is correlated with poorer health in the controls but has no significant association with the ME/CFS cohort (Fig. 7a). PROS1, also known as Protein S, is a well-known regulator of hemostasis, with important anti-coagulant effects [62]. The fact that it has no correlation with health of ME/CFS patients may reflect disturbed control of hemostasis in the disease.

Higher levels of CETP, Cholesteryl Ester Transfer Protein, are associated with increased fatigue on the MFI-20 (Fig. 7a). This protein controls the exchange of cholesteryl esters and triglycerides between HDL and low-density lipoproteins (LDL), and higher CETP would be expected to result in a less favorable LDL/HDL ratio, which is associated with heart disease [63]. Immune cells in ME/CFS patients have been observed to exhibit altered fatty acid oxidation, which could be related to differences in plasma fatty acid composition [64].

Higher levels of SERPINA5 were associated with better scores on the SF-36 general health and social functioning scales (Fig. 7b). SERPINA5 is a secreted serine protease inhibitor whose functions are not completely understood [65]. It was originally identified as an inhibitor of the anticoagulant protease-activated protein C [66]. While this fact suggests that higher SERPINA5 might increase coagulation, an in vitro study demonstrated that SERPINA5 can serve as both an anti-coagulant and a pro-coagulant depending on the presence of thrombomodulin [67]. Platelets contain SERPINA5 mRNA and can also take up the protein from the external milieu [68]. Our finding of a correlation of ME/CFS health status with a protein involved in hemostasis may be relevant to the recent findings of activated platelets and microclots in ME/CFS [69], as well as altered platelet gene expression profiles [52]. Furthermore, variants in the SERPINA5 gene have previously been associated with ME/CFS [70].

Correlations with several proteins were detected through robust linear regression that were not found through Spearman correlation (Table 3). CSF3, also known as Granulocyte colony-stimulating factor, increases with age in the ME/CFS cohort but is lower with age in the total cohort, perhaps indicating an inflammatory state in the patient cohort. EV chemokine CXCL1, which attracts neutrophils to regions of infection or injury, decreases with age in the total cohort. PFN1, profilin-1, which regulates actin polymerization, is predicted to be higher in males in the total cohort but lower in males with ME/CFS.

We used machine learning classifiers to identify proteins that discriminate between cases and controls. Previously, the proteomics dataset had been subjected to a similar analysis using LASSO, Random Forest, and XGBoost [14]. Seven proteins are common to the top 20 lists of all three machine learning methods. In addition to EV IL2, there were CAMP, IGLV1-47, CRTAC1, LRG1, IGF1, and TUBA1. Four of these were also in the group of 8 proteins that were common to the three methods in the prior study which analyzed only the plasma proteomics data [14]. IGF1 and TUBA1ABC were not in the top 20 when the total cohort was considered in the prior study. Among the seven common proteins, only EV IL2 and CAMP (Cathelicidin AntiMicrobial Protein) were increased in cases vs controls, and both are pro-inflammatory. The significance of a reduction in ILGVI-47 (Immunoglobulin Lambda Variable 1–47) in cases is difficult to predict but could reflect some unknown genotypic effect on susceptibility to ME/CFS. CRTAC1 (Cartilage Acidic Protein 1) is an extracellular matrix protein of unknown function, but improved growth of dermal fibroblasts in vitro, so lower levels could be detrimental. LRG1 (Leucine Rich Alpha-2-Glycoprotein 1) is secreted from hepatocytes and neutrophils, and higher levels are associated with beneficial functions (promoting wound healing) but also with a variety of diseases; thus, the significance of its reduction is unknown [71]. Lower levels of IGF1 (Insulin Like Growth Factor 1) are likely to be unfavorable for health, given its growth-promoting properties and effects on metabolism [72, 73]. TUBA1A, TUBA1B, and TUBA1C genes encode tubulin, an essential component of the cytoskeleton [74]. Tubulin signaling has been found to be disrupted following chemotherapy and is hypothesized to have a role in the neurocognitive impairment that often results following treatment [75].

EV-located IL2 is found in all three lists. IL2 was the only EV cytokine to distinguish cases and controls at q < 0.05 (Fig. 3). In our prior pilot study of EV cytokines in 35 cases and 35 controls, we did not find any significant difference in IL2 between cohorts [25]. Other EV cytokines that featured in the top 20 are VEGF, NGFB, IL15, CXCL8, CXCL10, CCL5, and CCL7, although VEGF, IL15, and CXCL10 did not discriminate cases and controls at q < 0.2, according to Wilcoxon tests (Fig. 3). Plasma cytokines IL7, TNFα, IL12p70, and IL22 were included on one or two of the top 20 lists. While no significant differences in any plasma cytokines were detected following correction for multiple testing, before correction TNFα was increased in cases at p = 0.016 [26]. Previously, Hornig et al. [41], who performed a larger study, with 298 cases and 348 controls, did not find significant differences between cases and controls for these cytokines. The cytokine profiling literature in ME/CFS has not resulted in consistent conclusions regarding altered cytokine levels between ME/CFS and controls.

This work does have some limitations. First, our study has a small sample size, especially given the heterogeneity of the symptoms of the illness and when measuring a large number of variables. The robustness of our findings needs to be verified in more diverse and larger cohorts.

Although ME/CFS has a higher disease burden in females [76] and an increasing number of sex differences in its pathophysiology have been discovered recently [77, 78], we were unable to report disaggregated sex data in our study due to sample size limitations (8 and 9 males compared to 41 and 40 females for the control and ME/CFS populations, respectively). Therefore, statistical comparisons between sexes were not feasible in our current study.

This study examined only peripheral blood and did not analyze other compartments such as cerebrospinal fluid. However, despite a small sample size, abnormalities in proteins of ME/CFS patients have been identified in cerebrospinal fluid studies [9, 46, 79]. Future proteomic research on peripheral blood of ME/CFS patients should strive to establish correlations with these findings.

Here, cytokine measurement in plasma and EVs was performed using different multiplex assays. Specifically, a 61-plex from Affymetrix was used to analyze cytokines in plasma samples, whereas a 48-plex from Biorad was used to measure cytokine content in EVs.

We opted for a precipitation method for EV isolation due to limited sample volumes (500 μl) and to enable analysis of the complete EV population. Using precipitating reagent ExoQuick tends to yield lower purity for EV isolated fractions compared to other methods such as ultracentrifugation and size exclusion chromatography. Future studies comparing these methods in cytokine analysis will be informative to ensure our results are reproducible using other EV isolation methods. Furthermore, EVs were not separated into different fractions by size or by the presence of particular surface molecules to allow analysis of these fractions separately. It is certain that distinct patterns will arise indicating the selective packaging of specific proteins into specific EVs.

It should be noted that the correlations reported in this study do not indicate cause-effect relationships, and further research is required to establish causality. For instance, since the diet of the subjects was not controlled in this study, discrepancies in cytokine profiles between different groups could be attributed to differences in their diets [80–82]. Thus, we cannot rule out the possibility that dietary factors may have influenced our results.

Ultimately, since this study employed a cross-sectional approach; examining longitudinal changes in EVs would require further exploration. Moreover, one-time sample collection prevents determining whether associations between symptoms and protein profiles in plasma and EVs of ME/CFS patients stem from disease progression. Future research is crucial to establish whether patients with ME/CFS consistently exhibit a specific cytokine signature and disease severity classification over time, or if these factors fluctuate.

## Conclusions

This work demonstrates the importance of collecting clinical data to determine whether particular molecules are correlated with the subjects’ conditions, allowing conclusions to be drawn about them even if their median values differ little between cases and controls. We have again demonstrated that cytokine/chemokine signaling networks in the circulation are altered between ME/CFS cases and controls. Finally, we have identified 20 proteins whose levels provided very high sensitivity and specificity for distinguishing ME/CFS and control samples. A more manageable subset of 7 of the 20 proteins still allows considerable separation of patients from controls (AUROC = 0.891, Fig. 8). These findings await confirmation in a larger dataset to determine whether they can be clinically useful for diagnosis or monitoring response to treatment.

## Supplementary Information

**Additional file 1: Figure S1**. PCA analyses for site and season for the three datasets examined. EV cytokines (**a**, **b**), plasma cytokines (**c**, **d**), and plasma proteomics (**e**, **f**). **Figure S2:** Correlogram of plasma cytokines and EV cytokines with |r| ≥ 0.6. “p” for plasma and “ev” for extracellular vesicles. **Figure S3 **Cross-Validated (5 fold, repeated 250 times) confusion matrices for distinguishing ME/CFS from controls with **a** the top 20, **b** the top 8 and **c** the top 7 proteins common to all three classifiers (entries are average percentages).

**Additional file 2.** p-values, q-values, and the ratios of mean protein analyte level for the ME/CFS group versus controls for the EV cytokine, plasma cytokine and plasma proteomics datasets. p-values are shown prior adjusting for multiple hypotheses and after correction for multiple comparison using the Benjamini–Hochberg method for false discovery rate (q-values).

## Data Availability

Data for extracellular vesicle size, quantification, and cytokine content is available on request to the authors. The mass spectrometry proteomics data have been deposited to the ProteomeXchange Consortium via the PRIDE partner repository with the dataset identifier PXD016622.

## Abbreviations

ME/CFS: Myalgic encephalomyelitis/chronic fatigue syndrome
EVs: Extracellular vesicles
NTA: Nanoparticle tracking analysis
PCA: Principal component analysis
SF-36: Short form 36 health survey
MFI-20: Multidimensional fatigue inventory scale
AUC: Area under the curve
BMI: Body mass index
IBS: Irritable bowel syndrome
LASSO: Least absolute shrinkage and selection operator
FDR: False discovery rate
AUROC: Area under the receiver operating characteristic curve
