# Supplementary material for: Proteomics and cytokine analyses distinguish myalgic encephalomyelitis/chronic fatigue syndrome cases from controls
Source: J Transl Med. 2023 May 13;21:322. doi: 10.1186/s12967-023-04179-3 (PMC10182359; doi:10.1186/s12967-023-04179-3)
Supplement: Supplementary file 1 — Additional file 1: Figure S1. PCA analyses for site and season for the three datasets examined. EV cytokines (a, b), plasma cytokines (c, d), and plasma proteomics (e, f). Figure S2: Correlogram of plasma cytokines and EV cytokines with |r| ≥ 0.6. “p” for plasma and “ev” for extracellular vesicles. Figure S3 Cross-Validated (5 fold, repeated 250 times) confusion matrices for distinguishing ME/CFS from controls with a the top 20, b the top 8 and c the top 7 proteins common to all three classifiers (entries are average percentages). [file 12967_2023_4179_MOESM1_ESM.docx]

Figure S1: PCA analyses for site and season for the three datasets examined. EV cytokines (a) (b), plasma cytokines (c) (d), and plasma proteomics (e) (f).


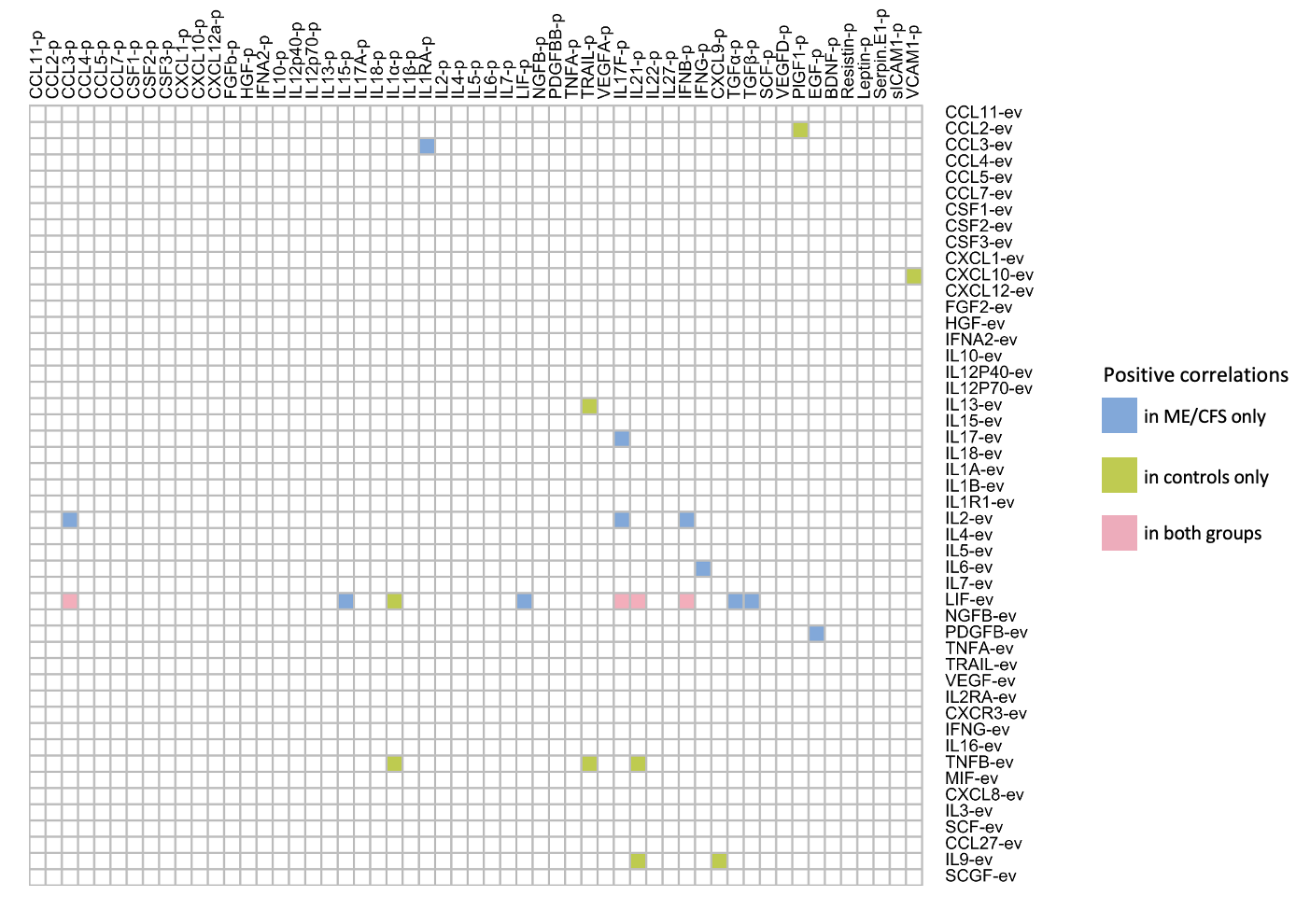


**Figure S2:** Correlogram of plasma cytokines and EV cytokines with |*r|* ≥ 0.6. “p” for plasma and “ev” for extracellular vesicles


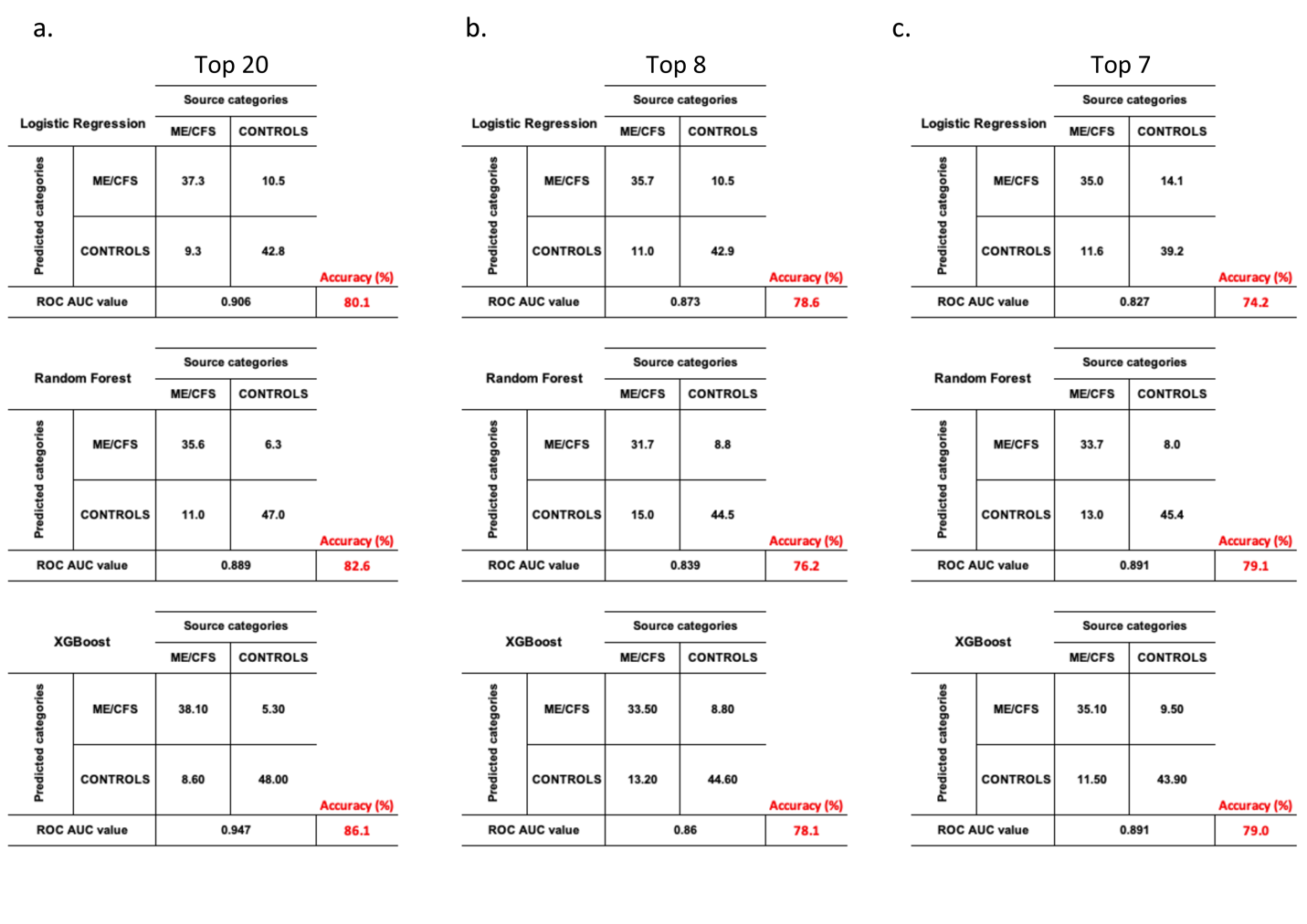


**Figure S3:** Cross-Validated (5 fold, repeated 250 times) confusion matrices for distinguishing ME/CFS from controls with (a) the top 20, (b) the top 8 and (c) the top 7 proteins common to all three classifiers (entries are average percentages).
